# Combined Effects of Moderate Hypoxia and Sleep Restriction on Mental Workload

**DOI:** 10.3390/clockssleep6030024

**Published:** 2024-07-23

**Authors:** Anaïs Pontiggia, Pierre Fabries, Vincent Beauchamps, Michael Quiquempoix, Olivier Nespoulous, Clémentine Jacques, Mathias Guillard, Pascal Van Beers, Haïk Ayounts, Nathalie Koulmann, Danielle Gomez-Merino, Mounir Chennaoui, Fabien Sauvet

**Affiliations:** 1 Armed Forces Biomedical Research Institute (IRBA), 91220 Brétigny-sur-Orge, France; anais.pontiggia@gmail.com (A.P.); haik.ayounts@gmail.com (H.A.);; 2 URP 7330 VIFASOM, Université Paris Cité, 75004 Paris, France; 3 École du Val-de-Grâce (EVDG), 75005 Paris, France; 4 Laboratoire Theresis, THALES SIX GTS, 91190 Palaiseau, France

**Keywords:** hypoxia, sleep restriction, MATB-II, auditory oddball, ECG, eye tracking, breathing rate, SpO_2_

## Abstract

Aircraft pilots face a high mental workload (MW) under environmental constraints induced by high altitude and sometimes sleep restriction (SR). Our aim was to assess the combined effects of hypoxia and sleep restriction on cognitive and physiological responses to different MW levels using the Multi-Attribute Test Battery (MATB)-II with an additional auditory Oddball-like task. Seventeen healthy subjects were subjected in random order to three 12-min periods of increased MW level (low, medium, and high): sleep restriction (SR, <3 h of total sleep time (TST)) vs. habitual sleep (HS, >6 h TST), hypoxia (HY, 2 h, F_I_O_2_ = 13.6%, ~3500 m vs. normoxia, NO, F_I_O_2_ = 21%). Following each MW level, participants completed the NASA-TLX subjective MW scale. Increasing MW decreases performance on the MATB-II Tracking task (*p* = 0.001, MW difficulty main effect) and increases NASA-TLX (*p* = 0.001). In the combined HY/SR condition, MATB-II performance was lower, and the NASA-TLX score was higher compared with the NO/HS condition, while no effect of hypoxia alone was observed. In the accuracy of the auditory task, there is a significant interaction between hypoxia and MW difficulty (F_(2–176)_ = 3.14, *p* = 0.04), with lower values at high MW under hypoxic conditions. Breathing rate, pupil size, and amplitude of pupil dilation response (PDR) to auditory stimuli are associated with increased MW. These parameters are the best predictors of increased MW, independently of physiological constraints. Adding ECG, SpO_2_, or electrodermal conductance does not improve model performance. In conclusion, hypoxia and sleep restriction have an additive effect on MW. Physiological and electrophysiological responses must be taken into account when designing a MW predictive model and cross-validation.

## 1. Introduction

In operational high-pressure situations, like those faced by pilots, rapid multi-information, task processing, and real-time decision-making are essential and need high mental workload capabilities. Mental workload (MW) can be characterized as the interaction between the components of the machine and task on the one hand and the operator’s resource capabilities, motivation, and state of mind on the other hand [1]. High and low MW are considered a potential factor in reduced pilot performance and increased accident risk [2,3]. Given the importance of assessing pilots’ mental workload for flight safety and efficiency, a number of studies have been carried out on monitoring this workload in complex real-life situations [4]. Several studies have thus explored it through subjective measures, cognitive performance assessments, and physiological indicators [5]. Quantitative assessment of cognitive performance involves analyzing central nervous system (CNS) signals like electroencephalogram and electrooculogram, as well as peripheral nervous system metrics such as heart rate, autonomous nervous system (ANS), heart rate variability (HRV), electrodermal activity, and/or electromyography [6].

Nevertheless, particularly under operational conditions, pilots are often faced with MW combined with physiological challenges, such as hypoxia and sleep restriction [7,8]. In addition, we know that higher-order cognitive functions require more energy and, therefore, more oxygen (O_2_), as this regulates all physiological and cognitive processes [9,10]. During flight, exposure to high altitude environments (>10,000 feet/3048 m) compromises the availability of oxygen (O_2_) due to decreased partial pressure of O2 (PO_2_) in atmospheric air, and this is potentially deleterious for cognition [7,11,12]. Correcting the O2 deficit requires the autonomic nervous system (ANS) to respond quickly and sufficiently to preserve the integrity of cognitive function.

We recently pointed out that sleep loss, which is also a condition often encountered by pilots in real-life situations, can impact physiological and cognitive responses to hypoxia [12]. In particular, a significant interaction between sleep deprivation and altitude (simulated by gas mixtures, 3810 m) has been described, which was reinforced by increasing workload in a complex performance task [13]. When subjects were deprived of sleep (one night), performance was significantly lower, and the greatest decrease in performance occurred at altitude. It, therefore, seems relevant to limit sleep deprivation before exposure to environmental hypoxia or even to create a sleep capital for oneself to preserve cognitive function [14].

Regarding MW, its accurate estimation requires a comprehensive approach taking into account individual and environmental factors [15,16,17]. The Multi-Attribute Task Battery (MATB)-II, a widely used multitasking flight simulation software package, is used to evaluate flight performance by simulating tasks encountered during actual flights, including flight operations, instrument monitoring, and emergency management. The MATB-II is used for workload assessment in low and high mental workload (LMW and HMW) modes. It is used for multitasking as well as for MW assessment [18]. The advantage of choosing MATB-II over other tests is its qualification to provide both objective and subjective scoring, as well as different levels of workload [19]. MATB-II has been used in studies with civilian [20] and military pilots (F-117A and helicopter) [21,22] and is considered relevant for assessing pilot workload and performance deterioration during prolonged wakefulness, diving, or exposure to hypoxia [23,24,25]. A recent study observed that exposure to normobaric-hypoxia (breathing 14.0% O_2_) activates the autonomic nervous system in military personnel during simultaneous exposure to high cognitive load (Enhanced AF-MATB simulator) [26].

The aim of this study was to assess the combined effect of moderate hypoxia (F_I_O_2_ = 13.6%, ~3500 m) and sleep restriction (<3 h vs. >6 h TST) on performance on the MATB-II tracking task at three MW levels (low, medium, high). We assessed the cardiovascular, respiratory, electrodermal, and pupil responses and correlations with parameters of MATB performance and subjective mental workload. In a second step, we evaluated the effects of physiological stress (hypoxia and/or sleep restriction) on correlations between physiological parameters and MATB-II performance and subjective workload. To assess the weight of physiological parameters in predicting the level of mental workload, ordinal logistic regression was performed. The overall aim is to propose robust predictors of periods of mental overload.

## 2. Results

Seventeen subjects completed the entire protocol. The mean age was 30.9 ± 7.4 years, and the mean BMI was 23.8 ± 1.7 kg/m^2^. The mean total sleep durations (TSTs) during sleep restriction conditions were 2.42 ± 0.15 h (SRHY) and 2.38 ± 0.23 h (SRNO) and during habitual sleep conditions were 6.5 ± 0.44 h (HSHY) and 6.4 ± 0.32 h (HSNO). The mean sleep efficiency index (SEI) (SEI = TST/TIB), an index of sleep quality, was equal to 84.2 ± 32.1%, without difference between conditions. 

### 2.1. Subjective Scale (NASA-TLX)

**Figure 1:**
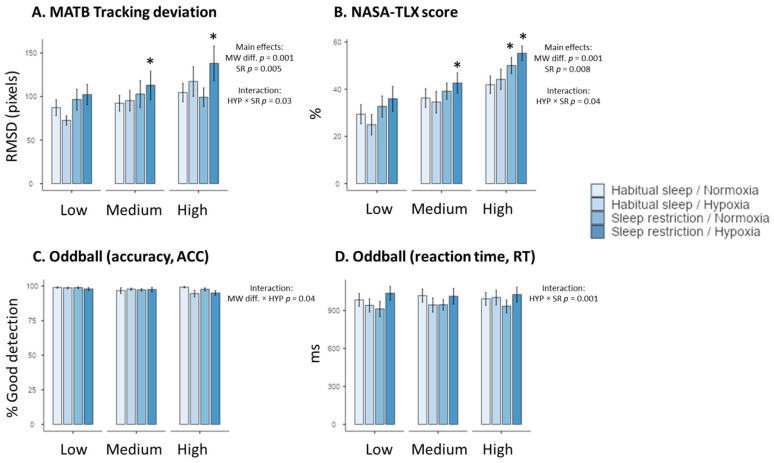
Performance to the MATB-II tracking task (**A**), NASA-TLX subjective scores (**B**), and accuracy (ACC) and reaction time (RT) to the auditory task ((**C**) and (**D**), respectively) in the four experimental conditions (Habitual sleep/Normoxia, Habitual sleep/Hypoxia, Sleep restriction/Normoxia, Sleep restriction/Hypoxia) and at the three MW difficulty levels (Low, Medium, High) * is a significant difference with the Habitual sleep/Normoxia condition, *p* < 0.05).

We showed an increase in the NASA-TLX global score with the difficulty (F_(2–176)_= 31.07, *p* < 0.001, η^2^ = 0.14, +40.1 ± 5.2% between high and low MW) and sleep restriction (F_(1–176)_ = 7.23, *p* = 0.008, η^2^ > 0.10, +12.1 ± 4.2% between high and low MW) (Table 1). There was an interaction between hypoxia and sleep restriction (F_(2–176)_= 3.47, *p* = 0.04, η^2^ > 0.03) (Table 1). The highest NASA-TLX score value was observed in the hypoxia + sleep restriction condition in high mental workload level (Figure 1), with a significant difference with the normoxia + habitual sleep condition (*p* = 0.01).

### 2.2. MATB-II Performance

Tracking performance decreased (increased RMSD values) with MW task difficulty (F_(2, 176)_ = 7.38, *p* = 0.001, η^2^ = 0.14, +25.4 ± 8.2%), and sleep restriction (F_(2–176)_= 8.12, *p* = 0.005, η^2^ = 0.06) (Table 1). We observed an interaction between hypoxia and sleep condition (F_(2–176)_ = 4.68, *p* = 0.03, η^2^ = 0.03) (Table 1). The highest RMSD value has been observed in the hypoxia + sleep restriction condition at the high MW level (Figure 1), with a significant difference with the normoxia + habitual sleep condition (*p* = 0.03). No significant effect (*p* = 0.05, η^2^ = 0.01) of hypoxia alone was observed.

No main effect of MW task difficulty, hypoxia, or sleep restriction was observed on auditory alarm detection accuracy and reaction time (RT) (Table 1, Figure 1). For accuracy, we observe a significant interaction between MW difficulty and hypoxia (F_(2–176)_= 3.14, *p* = 0.04, η^2^ = 0.03), with lower values at high MW under hypoxic conditions. We also showed a significant interaction between hypoxia and sleep restriction for the reaction time (F_(1–175)_ = 16.49, *p* = 0.001, η^2^ = 0.14), but no significant post hoc values were observed.

### 2.3. Heart Rate and Heart Rate Variability 

**Figure 2:**
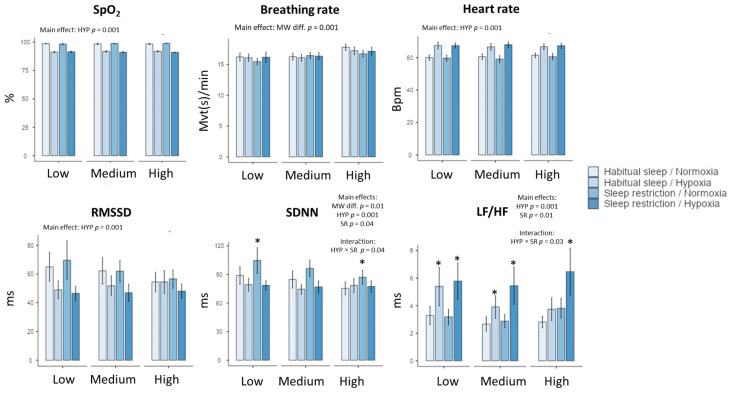
Changes in peripheral oxygen saturation (SpO_2_), respiratory (breathing rate), and cardiac parameters (heart rate and heart rate variability parameters) in the four experimental conditions (Habitual sleep/Normoxia, Habitual sleep/Hypoxia, Sleep restriction/Normoxia, Sleep restriction/Hypoxia) and at the three MATB-II MW difficulty levels (Low, Medium, High) * is a significant difference with the Habitual sleep/Normoxia condition, *p* < 0.05).

As expected, heart rate (HR) increased during MATB-II in hypoxia in comparison to normoxia (from 60.2 ± 0.75 to 67.15 ± 0.74 bpm, F_(1, 175)_ = 210, *p* < 0.001, with a large effect size, η^2^ = 0.20) (Figure 2). However, we observed no significant effect of MW task difficulty, sleep restriction, and no interactions (Table 2, Figure 2). For LF/HF, the cardiac index of sympathetic balance, we observed main effects of hypoxia (F_(1, 175)_ =29.52, *p* = 0.001, η^2^ = 0.18), sleep restriction (F_(1–175)_ = 6.33, *p* = 0.01, η^2^ = 0.06) and interaction between the two conditions (F_(1, 175)_ = 5.04, *p* = 0.03, η^2^ = 0.03) (Table 2, Figure 2). We didn’t show a MW task difficulty effect for LF/HF values. 

A main significant effect of MW task difficulty is shown for the indexes of global HR variability (SDNN, CVNN), CVI, and Shannon entropy. In addition, SDNN was impacted by hypoxia and sleep restriction, with significant interaction between the latter two factors (Table 2, Figure 2). However, the effect size for hypoxia is higher than MW task difficulty and sleep restriction (i.e., for SDNN, η^2^ = 0.14 for hypoxia, η^2^ = 0.06 for MW task difficulty, and η^2^ = 0.03 for sleep restriction). See the Appendix A Table A1 for abbreviations details. 

### 2.4. Respiratory Activity

The MW task difficulty had an impact on respiratory parameters independent of hypoxia or sleep restriction. Indeed, increased MW induced an increase in the mean respiratory rate (F_(1, 175)_ = 10.59, *p* = 0.001, η^2^ = 0.12), from 15.2 ± 0.31 for the low level to 17.34 ± 0.29 movements per minute for the high level (Table 2, Figure 2). We also observed an effect of MW difficulty on expiration duration and the HFn (normalized high frequency) component. Moreover, no significant effects of hypoxia and sleep restriction were observed, nor were any interactions observed, except for the hypoxia effect on respiratory amplitude (*p* = 0.03) (Table 2).

### 2.5. SpO_2_

As expected, hypoxia induced a significant decrease of SpO_2_, without interactions with sleep restriction or MW task difficulty. During MATB-II under hypoxia, SpO_2_ was 91.6 ± 0.3% (vs. 98.4 ± 0.3% in normoxia), without difference between habitual sleep and sleep restriction conditions or interaction with MW task difficulty (Table 2, Figure 2).

### 2.6. Electrodermal Activity

We showed no effect of MW workload difficulty, hypoxia, or sleep restriction on electrodermal activity parameters (Table 2).

### 2.7. Eye Tracking

**Figure 3:**
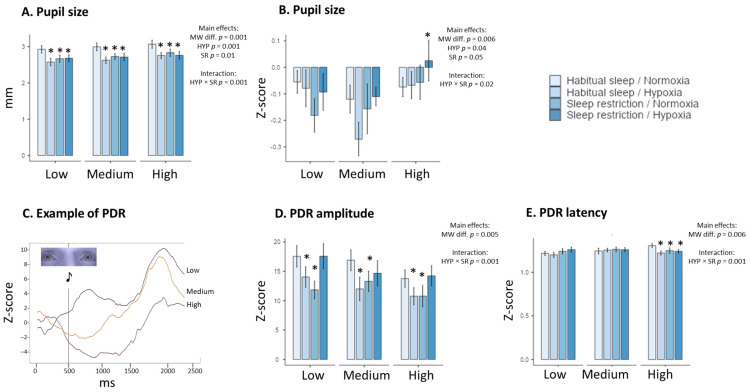
Changes in physiological Eye tracking parameters in the four experimental conditions (Habitual sleep/Normoxia, Habitual sleep/Hypoxia, Sleep restriction/Normoxia, Sleep restriction/Hypoxia) and at the three MATB-II MW difficulty levels (Low, Medium, High). Pupil size in raw values (**A**), pupil size in Z-score (**B**), an example of the Pupil Dilatation Response (PDR) at the three MATB-II MW difficulty levels (**C**), amplitude and latency ((**D**) and (**E**), respectively) of PDR. * is a significant difference with the Habitual sleep/Normoxia condition, *p* < 0.05).

A main MW task difficulty is observed on the pupil size in raw value (F_(2, 176)_ = 9.23, *p* = 0.001, η^2^ = 0.09 and for the Z-score, F_(2, 176)_ = 5.52, *p* = 0.006, η^2^ = 0.06), on the PDR amplitude (F_(2, 176)_ = 5.60, *p* = 0.005, η^2^ = 0.06 for the Z-score) and PDR latency (F_(2, 176)_ = 5.25, *p* = 0.006, η^2^ = 0.06 for the z-score) (Table 3, Figure 3). The main significant effects of hypoxia and sleep restriction have been observed in pupil size in raw value and the Z-score, with significant interaction between the two conditions (Table 3). We also observed a main MW task difficulty on the Z-score for PDR amplitude, latency (raw value and the Z-score), and time to return, with significant interaction between hypoxia and sleep restriction for the PDR amplitude (raw value and the Z-score), latency (the Z-score) and time to return (Table 3). An example of the PDR profile for one subject is illustrated in Figure 3C. 

### 2.8. Correlations (Pearson)

**Figure 4:**
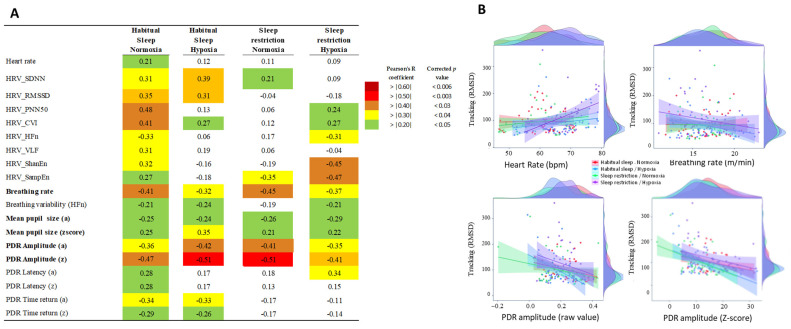
(**A**). Correlation analysis (with Pearson coefficient, R and P) between physiological parameters and MATB-II tracking performance in the four experimental conditions (Habitual sleep/Normoxia, Habitual sleep/Hypoxia, Sleep restriction/Normoxia, Sleep restriction/Hypoxia). Only parameters showing a significant correlation (corrected *p* < 0.05) with MATB-II tracking performance (RMSD value) in Habitual sleep/Normoxia were presented. *p* values take into account multiple comparison corrections [27] (**B**): examples of repeated-measures correlations between MATB-II tracking performance (RMSD values) and heart, breathing rate, and amplitude and Z-score of the PDR response in the four experimental conditions.

Correlations analyses showed that the breathing rate and pupil size in raw values significantly correlated (R > 0.20) with MATB-II tracking performance in all four conditions (Figure 4A). In addition, the breathing rate and pupil dilation response (PDR) amplitude (the raw values and Z-score) exhibited a higher level of correlation (R > 0.30) with tracking performance in all four conditions. The results also showed that the breathing rate and PDR amplitude (Z-score) significantly correlated (R > 0.20) with the NASA TLX score in three conditions and not in sleep restriction and hypoxia conditions (Supplementary Figure S1). Other physiological parameters were correlated with MATB-II tracking Performance in one or two conditions. In Figure 4B, we showed an example of one parameter (heart rate) that is significantly correlated with tracking performance only during habitual sleep and normoxia and three examples (breathing rate and PDR amplitude in raw and Z-score values) of robust significant correlations in the four conditions 

### 2.9. Ordinal Logistic Regression

Ordinal regression analysis confirmed that eye tracking and breathing parameters were the best predictors of increased mental workload, independently of physiological constraints (Table 4). Eye tracking (ET) (*p* = 0.01, model 1) or breathing (Br) (*p* = 0.02, model 4) parameters significantly predicted workload level. EDA and ECG parameters alone (models 2 and 3) did not predict the mental workload level. The combined ET and Br parameters (model 5) have the higher fit measures and p values (Table 4, *p* < 0.001). Additional ECG, SpO_2_, or EDA parameters (models 6, 7, 8, and 10) did not improve the model fit. 

Inside the model 5, the best predictors are the PDR amplitude (the Z-score) (*p* = 0.02) and the breathing rate (*p* = 0.02) (Table 5).

## 3. Discussion

In this work, we describe for the first time that moderate hypoxia combined with sleep restriction increases subjective mental workload using NASA TLX and decreases performance to the tracking task of the multitasking MATB-II, whereas the isolated impact of hypoxia or sleep restriction is low. Secondly, certain responses of ocular and respiratory parameters to increased mental load are still observed during hypoxia or sleep restriction and may be considered relevant predictors of mental workload.

There is no validated standard for the design of a MATB simulator inducing a low or high mental workload task (rate of stimuli, duration, additive task, etc.). Nevertheless, the MATB simulator levels have been validated compared to decreased behavioral MATB performance [2] and increased subjective workload score [2,28], as we observed in our work. In our experimental protocol, we increased mental workload by higher stimuli rate events and overlap, as described in previous studies, particularly under physiological constraints (hypoxia or sleep restriction) [21,29].

Moreover, increased MATB mental workload has been associated with changes in different electrophysiology indexes of mental workload [28,30] and fMRI responses [31]. The brain regions captured collectively in this fMRI study are key components involved in multitasking, such as planning, adjusting, and maintaining task information [31]. Nevertheless, it is very hard to extrapolate the specific cognitive functions altered during increased mental workload related to the multitasking MATB simulator. Among the various qualitative models developed by psychologists inherent to multitasking [32], working memory is probably the most salient parameter due to its predominant role in mental cognitive activities such as decision-making, attention, and searching [33]. In addition to working memory, other parameters, including the number of tasks, difficulty of tasks, and switching rate, affect human performance in a multitasking process [34].

In our study, we didn’t observe any changes in the Tracking task performance of MATB-II during the hypoxia condition, which, however, corresponds to a moderate level of exposure (FIO_2_ = 13.6%, ~3500 m, SpO_2_ = 91.4%). Little is known about how multiple environmental stressors interact with MATB-II performance in complex environments (hypobaric chamber, sleep deprivation, etc.). Four-hour hypoxic exposition at 8000 ft (2438 m) and 10,000 ft (3048 m) [22] and at 13,000 ft (3962 m) [35] does not appear to impair MATB performance. Our study, conducted after 2 h of exposure to hypoxia with an equivalent at 3500 m (11,500 ft), confirms these results. However, we show that the combination of moderate hypoxia with sleep restriction (limited to 3 h TIB (time-in-bed)) decreases the MATB performance in comparison to normoxia and sleep restriction alone. 

In our study, the sleep restriction condition decreases MATB-II tracking performance and increases subjective mental workload. The MATB, with appropriate difficulty levels, has already been used to evaluate performance during prolonged wakefulness for aviation pilots [20]. Indeed, Lopez et al. (2012) [36] examined the performance of MATB and Psychomotor Vigilance Task (PVT) and a simulated flight every 3 h during 35 h of sleep deprivation. Performance declined on all tests after about 18–20 h of continuous sleep deprivation. However, during the first half of sleep deprivation, simulated flight performance was predicted by the MATB, whereas during the second half, simulated flight performance was predicted by the PVT and much less by the MATB. Our study confirms a recent multi-experiment study [37] in which one night of Total sleep deprivation (TSD) reduced executive functions, regardless of hypoxic state (i.e., normoxia and hypoxia conditions were tested by gas breathing). Interestingly, several studies of cognitive performance at a high altitude have reported a greater reduction in cognitive performance in people with poor sleep markers [38,39,40]. The exact neurophysiological mechanisms by which moderate hypoxia combined with sleep restriction affect cognitive performance have yet to be elucidated, although both stressors appear to cause suboptimal functioning of the prefrontal cortex [41,42], the main brain region associated with executive functions (e.g., inhibition, working memory, cognitive flexibility) [43]. As the prefrontal cortex is highly sensitive to stress [44], it is therefore possible that a similar underlying neurophysiological mechanism contributes to the hypoxia *plus* sleep restriction effect on MATB that we observe. 

Recently, Bottenheft et al. [35] investigated how cognitive performance, assessed using MATB-II, is affected by the combination of two stressors that are operationally relevant for helicopter pilots: heat load (28 °C) and hypobaric hypoxia (13,000 ft, 3962 m), in a randomized control study. They observed that mainly heat load caused a decline in cognitive MATB-II performance [35]. In their work, only an arithmetic subtask was sensitive to hypobaric hypoxia [35]. These results, and our own, confirm the interest in studying mental workload under different environmental and physiological constraints.

With regard to physiological responses that might predict mental workload, Miyake et al. [45] indicated several years ago that one unique parameter is not enough and instead proposed a multidimensional assessment of workload using principal component analysis to combine several physiological parameters, including subjective scores [45]. These authors examined several physiological responses, including the HRV, and showed that the LF/HF ratio may not be a useful index of mental workload and would require a more complicated spectral analysis [45]. In this study, the HF/LF ratio shows large intra-individual (i.e., test/retest) and inter-individual differences. It has been established that HRV is nonspecific to mental workload [6], bearing in mind that it can be affected by many mental states and that spectral analysis of HRV requires at least 5 min of artifact-free recordings [46]. Interestingly, a recent study demonstrated that multiple features of ECG signals improve the classification of mental workload, among them the RR interval feature of HRV and other three features, T and P wave power, QRS complex power, and Sample Entropy (SampEn) [47]. In our study, among time-domain measures of heart rate, SDNN, which is a marker of global HRV (sympathetic and parasympathetic activity), responds to MW difficulty, hypoxia, and sleep restriction, with no significant interaction between the three conditions. In comparison, the RMSSD, which is more influenced by parasympathetic activity, responds only to hypoxia. As far as the LF/HF ratio is concerned, the main effect of hypoxia and sleep restriction are the only significant ones, and their effect-sizes are higher than the effect size of mental workload (difficulty). 

While the HRV response is well established at extremely high altitudes (i.e., severely hypoxic exposure) [48], it has been shown that in mild hypoxia (i.e., between 8000 and 10,000 feet (2438 m and 3048 m), which military aircrews are exposed to in most of their missions), the LF of RR interval variability increased significantly with no change in HF, nor change in all indices of cardiac baroreflex function [49]. However, the simultaneous exposure to mild hypoxia (14% O_2_) and high cognitive load (i.e., individuals continuously performing aviation-relevant tasks (including MATB) for 125 min) was shown to elicit a greater ANS response [26]. Our results added for the first time that SDNN, a measure of the global HRV, responds to MW difficulty, and among the analyzed complex features of ECG signals, the parameter of the RR intervals dynamics, Shannon Entropy, is also influenced by MW difficulty, hypoxia and the combined condition of hypoxia and sleep restriction. 

In this study, we show that ocular and respiratory measures appear to have a stronger relationship with mental workload difficulty. This result is important for the design of robust predictive models of MW adapted to operational conditions. The ocular measures included the pupil size and the pupil dilation response to the auditory Oddball-like stimuli (the amplitude, latency, and time return parameters), with the magnitude of the pupil response being previously rated as greater for aural presentation than for visual presentation during cognitive activities [50]. Our results showed a main effect of MW difficulty on pupil size and the three components of the pupil dilation response, amplitude (expressed as the Z-score), latency, and return-to-baseline, with significant interactions between hypoxia and sleep restriction. 

The ocular measures can be used to discriminate between high and low mental task load, with blink rate (assessed using electrooculogram, EOG) decreasing during the more highly visually demanding segments of a 90-min flight scenario [51], and blink duration (assessed using EOG) decreasing with time-on-task during MATB [52]. The blink counts (assessed using video capture) were significantly different between the high and low task load conditions for the tracking MATB task and were significantly negatively correlated with the NASA-TLX scores across all dimensions [53]. With the method of our protocol (i.e., the use of the Eye Tracker), it was not possible to study the number of blinks because during the MATB, subjects frequently look at the keyword, and therefore, the signal is lost. Nevertheless, in future studies, the use of EOG to assess blinks would improve the overall multimodal physiological characteristics for monitoring mental workload [54]. In our study, the Z-score for the amplitude in the pupil dilation response correlated negatively with the RMSD measure of MATB tracking performance in all four experimental conditions (i.e., the higher RMSD measure, indicating poor tracking performance). This additional result underlines the interest in the pupil dilation response in the study of mental workload during a physiological constraint such as hypoxia combined with sleep restriction [15,55]. 

Respiratory changes have also been considered as an index of cognitive load, as reviewed by Grassmann et al. [9]. Among respiratory parameters, it is indicated that mentally demanding episodes are clearly marked by faster breathing and higher minute ventilation, while respiratory amplitude appears to remain rather stable. Our results show the main effect of MW difficulty in the MATB tracking task on breathing rate over all four experimental conditions. Interestingly, under assumed (unspecified) stable experimental conditions (a normoxic environment with habitual sleep), respiratory rate and NASA TLX score increased, and heart rate did not change as the task became more difficult in a computer simulation of air traffic control [56]. We have therefore confirmed and extended this previous work as we show that breathing rate and the amplitude of pupil dilation respond to MW difficulty in the tracking MATB task (and at NASA-TLX) under conditions of normoxia and moderate hypoxia with habitual and restricted sleep. This is illustrated by significant negative correlations between RMSD tracking performance (and NASA TLX) values, breathing rate, and the amplitude of pupil dilation response. Furthermore, our results indicated that MW difficulty influenced the high-frequency component (the normalized HFn) in the spectral analysis of the respiratory signal. Few studies have evaluated the spectral components of the respiratory signal during increased mental workload, with the exception of Veltman et Gaillard [57], who described respiration measured by means of inductive plethysmography during a simulated flight task. These authors showed that subjects tended to breathe deeper and more slowly just after landing, without differences between the other flight segments. More recently, for Vlemincx et al. [58], the total breathing variability increased during mental arithmetic load and decreased during sustained attention [58]). The ordinal logistic regression analysis we performed did not confirm that the spectral LFn and HFn components of the breathing variability can be considered predictors of increased mental workload.

With regard to the ordinal logistic regression analysis, we confirmed that two physiological parameters could predict MW difficulty in the MATB tracking task, the breathing rate and the amplitude of pupil response, under experimental conditions of exposure to moderate hypoxia combined or not with sleep restriction. Adding pulse oximetry (SpO_2_) and the EDA phasic and tonic components that were not sensitive to MW difficulty (the only significant effect was the SpO_2_ sensitivity hypoxic conditions) did not improve the logistic regression model. 

The main limitations of our study are the relatively small number of subjects and the experimental laboratory context, which limit extrapolation to the operational aeronautical situation. During complex simulated flight, the MATB-II tracking task (along with the SYSMON and RESMAN tasks) was shown to predict prospective memory performance in fifty-one pilots [59]. These authors also showed that (i) pilot level and hours flown did not correlate with any of the MATB-II subtests, (ii) recent pilot-in-command hours were negatively correlated with SYSMON errors in the medium-and high-difficulty levels, and (iii) the number of years licensed was positively correlated with SYSMON errors, although this may be an artifact of the negative effect of age on performance. All these results highlight the relevance of using the MATB simulator in an aeronautical context. Nevertheless, our results must be confirmed under different conditions (e.g., +Gz accelerations of the fighter pilot and/or thermal constraints in the laboratory or ecological settings, etc.), with a larger number of subjects, in order to lead to more robust predictive models. 

In future studies, it will be interesting to evaluate EEG parameters and their relationship with mental workload, with a view to a robust predictive model of mental workload. Indeed, recent technological advances have enabled the development of inexpensive and highly portable brain sensors with dry electrodes to monitor the “brain at work” in complex real-world situations such as aircraft operation [60]. However, it is necessary to compare these sensors in the real operational conditions of a fighter plane pilot, for example. Real-time monitoring of mental workload is a crucial step in building closed-loop adaptive support systems for human-machine systems. Estimators of mental workload based on spontaneous electrophysiological responses have shown great potential to achieve this goal.

## 4. Materials and Methods

### 4.1. Subjects

We included seventeen healthy men (20–45 years) with no history of contraindication to altitude exposure or high-altitude exposure in the past 3 months. We excluded subjects with sleep disturbances, verified by the Pittsburg sleep quality index >5 [61] or a usual TST < 6 h. The protocol was approved by the “Comité de protection des personnes (CPP) Ile de France VIII”, national IDRCB n°2022-A00464-39. All procedures were conducted in accordance with the Declaration of Helsinki, as revised in 2001. All participants provided written informed consent before participation. The study has been carried out at the French Armed Forces Biomedical Research Institute (IRBA, Brétigny-sur-Orge, France). It was part of a global study related to the physiological effect of a 5 h normobaric hypoxic exposure. See the Clinical trial database for the global description of the protocol (NCT055663688).

### 4.2. Mental Workload Tasks

#### 4.2.1. Multi-Attribute Task Battery (MATB-II)

**Figure 5:**
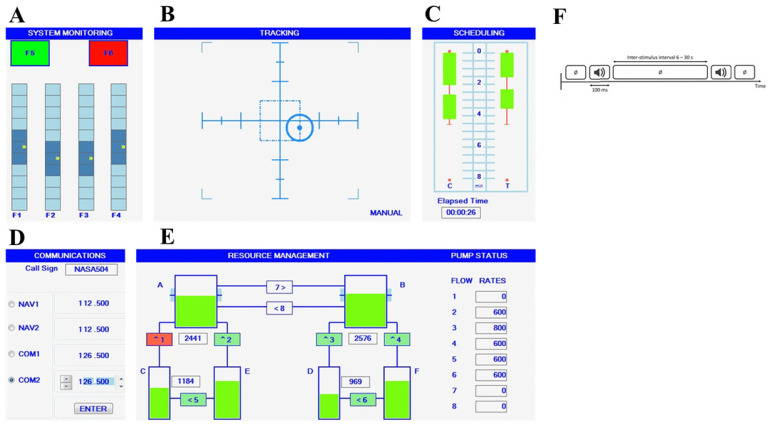
Illustration of the four subtasks of the Multi-Attribute Task Battery (MATB)-II and the auditory Oddball-like task: SYSTEM MONITORING (**A**) task in the upper left corner where participants had to respond as quickly as possible to scale fluctuations via keystrokes, TRACKING (**B**) task in the upper corner where participants had to keep a tracker as close to the center with a joystick, COMMUNICATIONS (**D**) task in the bottom left corner where participants had to only answer broadcast messages that matched their call signs and RESSOURCE MANAGEMENT (**E**) task in the bottom right corner that required participants to keep tanks’ levels as close to target level as possible (2500 for the left and 1000 for right) by managing eight pumps. AUDITORY ODDBALL-LIKE (**F**) task that requires ignoring frequent tone and detecting infrequent auditory stimulus. (**C**) A workload rating survey is not a task but an automatic evaluation of the temporal progression; no action is required.

The Multi-Attribute Task Battery (MATB) is a computer-based assessment simulator designed to evaluate an operator’s mental workload based on performance measures. Originally developed by NASA [62] and later re-issued on the Microsoft Visual Studio platform and Python in a free open version that we used in this study [63]. MATB-II is adapted for non-pilot participants and simulates tasks resembling those encountered by pilots during flights. The test includes four concurrent tasks: System Monitoring (SYSMON), Tracking (TRACK), Communications (COMM), and Resource Management (RESMAN), as illustrated in Figure 5: 

In the tracking (TRACK) task, subjects use a joystick to maintain a moving reticule (tracker) to the target center. Tracker dispersion remained consistent across all subjects and sessions. 

In the communications (COMM) task, subjects respond to recorded messages simulating air traffic control (ATC) communication. Randomly announced call signs and radio channels require participants to set the appropriate frequency, while non-target messages are ignored. Messages are delivered through earphones. 

In the resource management (RESMAN) task, subjects maintain fluid levels in two main tanks (Tanks A and B) replenished by sub-tanks (Tanks C, D, E, and F) through pumps. They control fuel flow by activating or deactivating numbered pumps using the corresponding keyboard number, adjusting to varying speed and force. Pump breakdowns and repairs occur during the session, adding complexity to the task.

In the system monitoring (SYSMON) task, subjects are involved in monitoring system status indicators with four scales and keys (F1–F4). They reset deviated indicators to the center using the corresponding keyboard keys. The performance analysis focuses on the tracking (TRACK) task, chosen for its consistency and continuous measurement across all difficulty levels. This performance measure provides a comprehensive evaluation of participants’ performance across MATB-II tasks. During the experiment, the X and Y positions of the tracker for the tracking task were recorded. Performance was assessed by calculating the Root Mean Square Deviation (RMSD) from the target center position, measured in pixel units (Equation (1)) [2].

(1) $$\mathrm{RMSD}=\sqrt{\frac{\sum_{i=1}^{n}\left({X_{i}}^{2}+{Y_{i}}^{2}\right)}{n}},$$

X s the position of the reticle in X axis in pixel; Y the position of the reticle in Y axis in pixel with (0,0) the center; and n the number of samples.

#### 4.2.2. Additional Auditory Task 

To acquire pupil dilation responses (PDR), subjects performed an additional oddball-like task during MATB-II simulator as previously described in previous research on mental workload [34,64,65,66] or real flights [60]. Auditory stimuli were presented binaurally through headphones with a novelty oddball paradigm, in which two types of stimuli, 500 Hz frequency tones (80%) as the standard stimuli and 2000 Hz infrequent tones (20%) as the deviant stimuli, were included. All stimuli were presented with an inter-trial interval of 5000 ms, allowing sufficient time for the pupil to return to baseline. 

Subjects were instructed to press the “space” key promptly upon hearing the deviant sound. The number of stimuli was consistent across all difficulty levels (144 stimuli per level). Task performance was assessed by calculating accuracy (% good detection) and average reaction time. 

#### 4.2.3. Mental Workload Levels

The difficulty levels (low, medium, and high) were determined by adjusting event frequencies and overlap for each sub-task, as detailed in Table 6. The order of the levels was counterbalanced across subjects. The effect of workload on performance was assessed by the tracking performance [2,67].

#### 4.2.4. Subjective Assessment

The subjective workload was assessed using the NASA Task Load Index (NASA-TLX) after each MATB level. The weighted average on six subscales from 0 (low) to 100 (high) is recorded: mental demand, physical demand, temporal demand, performance, effort, and frustration [19]. 

### 4.3. Electrophysiological Recording and Processing

ECG signals were recorded using a bio amplifier (FE232 Dual Bio Amp, ADInstruments, Sydney, Australia) with a 1000 Hz sampling rate. The signal quality was visually controlled with RR interval and artifacts (Labchart software, V8.1.2, ADInstruments, Dunedin, New Zealand). Analyses were conducted with the Python open-source tool Neurokit (Neurokit method) [68]. We investigated heart rate (beat per minute), heart rate variability (HRV), time, frequency, and information domains [69]. We selected some features that are modified by mental workload according to studies. These features are summarized in Table A1 (Appendix A). For the selected periods, the mean, the SD between normal-to-normal intervals (SDNN), and the root mean square of successive differences of successive normal-to-normal intervals (RMSSD) were calculated. SDNN is a measure of total HRV, and RMSSD corresponds to short-term variability [46]. The normalized high-frequency component of the HRV (HFn) provides an estimate of the vagal tone, whereas both sympathetic and vagal tones contribute to the normalized low-frequency component (LFn) [46]. The LF/HF ratio is considered a reflection of the sympatho-vagal balance or sympathetic modulation [46]. 

A respiratory chest belt sensor (TN1132/ST Respiratory Belt, ADInstruments, Sydney, Australia) was employed to record the subjects’ respiratory signals. The signal was recorded using the software Labchart, V8.1.2 with a 1000 Hz sampling rate. Analyses were conducted using the Python open-source tool Neurokit. We selected some features that were modified by the mental workload [9]. These features are summarized in Table A2 (Appendix A). In particular, we investigated breathing rate, expiratory and inspiratory durations, and frequency domain variables [57]. 

Pulse oximetry (SpO_2_) was continuously measured to estimate the level of arterial O_2_ saturation in the blood. Participants oxygen saturation was continuously assessed using a radical 7 Radical-7^®^ Pulse Oximeter (Masimo, Neuchâtel, Switzerland). The signal was recorded with a 1000 Hz sampling. The outliers (data < 80%) were automatically deleted and considered as non-valid.

An eye tracker Tobii 4C system (Tobii AB, Danderyd, Sweden) was used to record pupil diameter changes in both eyes at 90 Hz. Before each run, participants performed a 5-point calibration procedure. Using self-written Python code, data points that were contaminated by artifacts were replaced by linear interpolation. Next, we averaged across measurements from both eyes and applied a six-point moving average filter. Pupil size was expressed in raw value and Z-score [(raw value − mean baseline value)/baseline standard deviation]. Because the periods of signal loss exceeded 10% (in particular when the subject looked at the keyword), the calculus of the blink parameters was not possible. We also conducted a pupil size reactivity analysis after each oddball stimulus. For this analysis, the mean size in the 500 ms pre-stimulus baseline was extracted and used to correct the baseline for each trial, after which subject means were calculated. Based on the waveform shape, we then extracted the maximum dilation (in mm) and latency thereof (in ms) in a time window of 1000 to 1600 ms after the onset of the Oddball auditory stimulus, as well as the average dilation curve in a second-time window (1600–2000 ms) (called return-to-baseline, in mm) [70]. Parameters are described in the Appendix A, Table A3.

An Empatica wristband (E4) was employed to record the subjects’ electrodermal activity (EDA) signals. Electrodermal activity is derived from two sensors that constantly measure fluctuating changes in certain electrical properties of the skin. The output of the E4 includes a spreadsheet that contains one column in which SCL in micro Siemens at 4 Hz sample is specified. Analyses were conducted using the Python open-source tool Neurokit 0.2.1 for Python. The EDA signal was smoothed with a rolling filter of 500 data points per block. Several EDA features related to signal and peak intensity were analyzed [71]. They include the phasic component (also called EDA Skin Conductance Response) and the tonic component (also called Skin Conductance level) related to peak intensity (Appendix A, Table A4).

To synchronize electrophysiological recordings with MATB-II and oddball-like auditory tasks we used the Lab Streaming Layer libraries (LSL, Swartz Center for Computational Neuroscience, UCSD, November 2018). 

### 4.4. Normobaric Hypoxia Exposure

The experiment took place in a normobaric hypoxic chamber measuring 40 m^3^ (Sporting Edge, Basingstoke, UK). The chamber was designed to ensure constant brightness, temperature (21 °C), and a low level of ambient sound. In normoxia, the FIO_2_ was fixed at 21%, whereas in hypoxia, it was at 13.6%, creating an equivalent of 3500 m exposure. For the tests, participants were seated at approximately 70 cm from a 22′’ computer screen (1680 × 1250 pixels). Ambient luminance was of 200 lx. We previously controlled so that the opening of the chamber for the subject installation did not increase FIO_2_. The test was assessed after 2 h of exposure to normobaric hypoxia or normoxia. 

### 4.5. Sleep Conditions

On the week before admission to the sleep laboratory, all the participants were instructed to maintain regular sleep-wake behavior with their usual 7 to 8 h of sleep (i.e., in bed around 10 p.m. until 6 a.m.) and avoid late hours. In the habitual sleep condition, the participants have been asked to wake up at 6:00 a.m. and to respect an 8 h’ time in bed (TIB). In the sleep restriction condition, the participants arrived at the sleep laboratory the previous day (at 8:00 p.m.) and were then allowed to sleep 3 h (between 3:00 a.m. and 6:00 a.m.). The total sleep time was assessed using an accelerometer fitted in the non-dominant wrist (MotionWatch 8, CamNTech, Papworth Everard, UK). 

Before 3 a.m., participants were kept awake with a variety of activities. The sleep-restricted nights were in the same apartment, and an experimenter was present at all times to monitor the participants’ wakefulness.

### 4.6. Protocol

**Figure 6:**
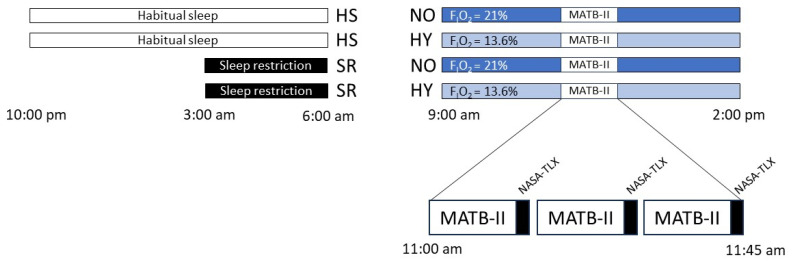
The study protocol. The order of conditions is: Habitual sleep Normoxia (HSNO), Habitual sleep Hypoxia (HSHY), Sleep restriction Normoxia (SRNO), Sleep restriction Hypoxia (SRHY). The levels of MATB-II difficulty (low, medium, or high) are randomized. Black square: NASA-TLX test.

Participants underwent a 15-min practice session of MATB-II within seven days prior to their experimental day. Instructions were reiterated before the start of their actual experiment to minimize any potential learning effects. Participants were crossover subjected to 4 experimental conditions with a wash-out period of at least 7 days after hypoxia or sleep restriction sessions (Figure 6):

- Normoxia, (NO, FIO_2_ at 21%) after a habitual night’s sleep (HS, >6 h TST) (HSNO).
- Normoxia, (NO, FIO_2_ at 21%) after a night of sleep restriction (SR, <3 h TST) (SRNO).
- Normobaric hypoxia (HY, FIO_2_ at 13.6%) after a habitual night’s sleep (HS, >6 h TST) (HSHY).
- Normobaric hypoxia (HY, FIO_2_ at 13.6%) after a night of sleep restriction (SR, <3 h TST) (SRHY).

On the day of the test, participants were placed in the normobaric hypoxic chamber for 5 h (from 9:00 a.m. to 2:00 p.m.). MATB-II simulator was assessed at 11:00 a.m., i.e., after 2 h of hypoxia or normoxia exposure. Participants completed in random order the three levels of difficulty of MATB-II: low, medium, and high, under the four different conditions. Each level lasted 13 min, with a 1-min baseline recording period at the start (MATB-II screen without stimuli) followed by a 12-min MATB-II + an auditory task. Following each level, participants completed the NASA-TLX scale. The experimental protocol is presented in Figure 6.

### 4.7. Statistical Analyses

The analysis of MATB-II performance focused solely on the main sessions, excluding the practice session. Statistical analyses have been conducted using a mixed linear model with the following factors: Hypoxia (two fixed levels, hypoxia vs normoxia), Sleep restriction (two fixed levels: sleep restriction vs. habitual sleep), MATB-II difficulty (ordinal factor, with three levels: low, medium and high), and a random factor for the subjects. The Satterthwaite method was used for the degrees of freedom calculation. Effect sizes were estimated with the calculation of the atrial eta square (η^2^ > 0.01 indicates a small effect, η^2^ > 0.06 a medium effect, and η^2^ > 0.14 a large effect). In the case of significant main effect or interaction, significant differences between conditions were identified using the Bonferroni T post-hoc test. 

Correlations between parameters were made using the Pearson coefficient. To manage the type 1 error by multiple comparisons, we used the Benjamini and Hochberg method [27].

To assess the weight of parameters for the prediction of workload level, a forward ordinal logistic regression [72] was made with the workload level in the dependent variable, sleep and hypoxia, and non-correlated parameters as predictors. Different models successively including features of ECG, SpO_2_, breathing rate, electrodermal conductance, and eye tracking were tested and compared. For each model, we removed collinear parameters and tested successive retrospective analyses to improve the performance. Analyses were made using Jamovi^®^ for R (version 1.6.15). The significance level of *p* < 0.05 was used for all. Results have been expressed as mean ± SEM (standard error of the mean). 

## 5. Conclusions

In healthy individuals, the combined exposure to moderate hypoxia and sleep restriction increased subjective mental workload and decreased performance to the MATB-II tracking task. Physiological and neurophysiological responses must be taken into account for the design of a predictive model of MW with cross-validations in different experimental conditions (hypoxia, sleep restriction, etc.) [17]. These responses will also help identify the exact mechanisms involved in the effects of moderate hypoxia with sleep restriction. Our results should be taken into account in future studies monitoring the mental workload associated with the different MATB-II tasks and other complex multitasks.

## Figures and Tables

**Table 1:** MATB-II performance and NASA-TLX score during experimental conditions, mixed linear model results.

Parameters	Difficulty F_(2–176)_	Hypoxia F_(1–175)_	Sleep Restriction F_(1–175)_	Diff. × Hyp. F_(2–176)_	Diff. × Sleep. F_(2–176)_	Hyp. × Sleep. F_(1–175)_	Diff. × Hyp. × Sleep. F_(2–176)_
NASA-TLX	**31.07 (0.001)**	1.56 (0.21)	**7.23 (0.008)**	0.26 (0.77)	0.32 (0.73)	**3.47 (0.04)**	1.20 (0.31)
Tracking (RMSD)	**7.38 (0.001)**	3.81 (0.05)	**8.12 (0.005)**	1.00 (0.37)	0.52 (0.60)	**4.68 (0.03)**	0.24 (0.79)
Auditory alarm (accuracy, ACC)	7.73 (0.06)	0.19 (0.66)	0.62 (0.43)	**3.14 (0.04)**	0.72 (0.49)	0.01 (0.98)	1.86 (0.16)
Auditory alarm (reaction time, RT)	3.30 (0.07)	3.10 (0.08)	0.02 (0.90)	0.13 (0.88)	1.62 (0.20)	**16.49 (0.001)**	0.40 (0.67)

Values are F (p); bold indicates the significant results. RT is reaction time, ACC is accuracy, RMSD is root mean square deviation, Diff. is the MATB difficulty level, Sleep is the sleep restriction condition (<3 h vs. >6 h TST), and Hyp. is the hypoxia condition (13.6 vs. 21% of F_I_O_2_).

**Table 2:** Physiological changes during experimental conditions, mixed linear model results.

Parameters		Difficulty F_(2–176)_	Hypoxia F_(1–175)_	Sleep Restriction F_(1–175)_	Diff. × Hyp. F_(2–176)_	Diff. × Sleep. F_(2–176)_	Hyp. × Sleep. F_(1–175)_	Diff. × Hyp. × Sleep. F_(2–176)_
**HR**		0.58 (0.56)	**210.0 (0.001)**	0.63 (0.43)	0.35 (0.70)	0.03 (0.97)	2.97 (0.09)	0.15 (0.86)
**HRV**	**RMSSD**	2.41 (0.09)	**18.40 (0.001)**	0.14 (0.71)	0.60 (0.55)	0.33 (0.72)	2.58 (0.11)	0.18 (0.83)
	**SDNN**	**4.65 (0.01)**	**13.05 (0.001)**	**4.52 (0.04)**	0.85 (0.43)	0.37 (0.69)	**4.41 (0.04)**	0.18 (0.84)
	**CVNN**	**4.73 (0.01)**	0.29 (0.59)	**7.26 (0.008)**	0.55 (0.58)	0.35 (0.70)	3.26 (0.07)	0.26 (0.77)
	**pNN50**	1.12 (0.33)	**49.42 (0.001)**	0.05 (0.82)	0.03 (0.97)	0.03 (0.97)	**6.59 (0.01)**	0.07 (0.93)
	**HTI**	**6.72 (0.01)**	1.51 (0.22)	**14.12 (0.001)**	0.72 (0.49)	0.48 (0.53)	**7.91 (0.01)**	0.27 (0.76)
	**CVI**	**5.28 (0.01)**	**25.80 (0.001)**	2.36 (0.13)	0.44 (0.64)	0.23 (0.80)	**6.07 (0.02)**	0.08 (0.93)
	**TINN**	3.04 (0.05)	**6.58 (0.01)**	3.13 (0.08)	1.15 (0.32)	0.84 (0.43)	**9.87 (0.002)**	0.20 (0.82)
	**HFn**	0.25 (0.78)	**9.83 (0.002)**	**25.78 (0.001)**	0.01 (1.00)	0.50 (0.61)	0.02 (0.88)	1.11 (0.33)
	**LFn**	0.52 (0.60)	**20.76 (0.001)**	0.18 (0.67)	0.15 (0.86)	1.13 (0.33)	**4.10 (0.05)**	0.46 (0.63)
	**VLF**	0.69 (0.50)	**11.08 (0.001)**	2.48 (0.12)	2.36 (0.10)	1.26 (0.29)	0.91 (0.34)	0.14 (0.87)
	**LF/HF**	0.46 (0.63)	**29.52 (0.001)**	**6.33 (0.01)**	0.24 (0.79)	2.05 (0.13)	**5.04 (0.03)**	0.25 (0.78)
**Entropy**	**SampEn**	1.03 (0.36)	**7.52 (0.007)**	**5.22 (0.02)**	0.41 (0.66)	0.06 (0.94)	0.81 (0.37)	0.07 (0.93)
	**ApEN**	1.23 (0.30)	1.27 (0.26)	**4.51 (0.04)**	0.21 (0.81)	0.06 (0.94)	0.24 (0.63)	0.11 (0.81)
	**ShanEN**	**6.47 (0.002)**	0.44 (0.51)	**14.63 (0.001)**	0.45 (0.64)	0.06 (0.94)	**7.03 (0.009)**	0.04 (0.96)
**RSP**	**Rate**	**10.59 (0.001)**	0.02 (0.90)	1.17 (0.28)	0.16 (0.85)	0.48 (0.62)	2.11 (0.15)	0.22 (0.80)
	**Amplitude**	0.65 (0.52)	**4.95 (0.03)**	2.39 (0.14)	0.84 (0.43)	0.08 (0.92)	1.24 (0.61)	1.59 (0.20)
	**Inspiration (dur.)**	2.89 (0.06)	0.31 (0.58)	0.16 (0.69)	0.17 (0.84)	0.37 (0.69)	0.01 (1.00)	0.27 (0.77)
	**Expiration (dur.)**	**6.10 (0.003)**	0.33 (0.57)	0.72 (0.40)	0.01 (1.00)	0.35 (0.71)	0.11 (0.74)	0.41 (0.67)
	**HFn**	**8.86 (0.001)**	0.53 (0.47)	0.78 (0.38)	0.62 (0.54)	0.92 (0.40)	2.28 (0.13)	0.51 (0.60)
	**LFn**	1.00 (0.37)	1.52 (0.22)	3.35 (0.07)	1.83 (0.16)	0.28 (0.76)	3.58 (0.06)	0.07 (0.93)
**SpO_2_**		0.09 (0.91)	**619.1 (0.001)**	0.56 (0.45)	0.02 (0.99)	0.02 (0.98)	1.34 (0.25)	1.12 (0.33)
**EDA**	**Phasic activity**	0.58 (0.56)	0.43 (0.51)	2.51 (0.12)	0.45 (0.64)	0.61 (0.55)	0.08 (0.78)	2.06 (0.13)
	**Tonic activity**	0.04 (0.96)	0.94 (0.33)	1.06 (0.31)	0.03 (0.97)	0.01 (0.99)	2.42 (0.12)	0.09 (0.91)

HR is heart rate, HRV is heart rate variability, RSP is respiratory parameters, dur. is duration, SpO_2_ is peripheral oxygen saturation, and EDA is electrodermal activity; all other abbreviations can be found in the Appendix A Table A1 and Table A2.

**Table 3:** Eye and pupil changes during experimental conditions, mixed linear model results.

Parameters	Difficulty F_(2–176)_	Hypoxia F_(1–175)_	Sleep Restriction F_(1–175)_	Diff. × Hyp. F_(2–176)_	Diff. × Sleep. F_(2–176)_	Hyp. × Sleep. F_(1–175)_	Diff. x Hyp. × Sleep. F_(2–176)_
**Pupil size**							
Raw size	**9.23 (0.001)**	**55.95 (0.001)**	**18.65 (0.01)**	0.92 (0.40)	0.15 (0.86)	**16.47 (0.001)**	0.08 (0.93)
Z-score	**5.52 (0.006)**	**4.10 (0.04)**	3.52 (0.05)	0.27 (0.76)	1.72 (0.10)	**5.12 (0.02)**	0.11 (0.89)
**Pupil dilation response (PDR)**							
Amplitude (r)	0.64 (0.53)	**4.03 (0.04)**	0.63 (0.43)	0.64 (0.29)	2.19 (0.12)	**14.24 (0.001)**	0.95 (0.29)
Amplitude (Z)	**5.60 (0.005)**	0.99 (0.32)	1.34 (0.23)	0.24 (0.79)	2.19 (0.12)	**20.61 (0.001)**	0.41 (0.66)
Latency (r)	**3.46 (0.03)**	0.27 (0.63)	1.79 (0.18)	2.20 (0.11)	2.11 (0.04)	4.06 (0.05)	1.92 (0.15)
Latency (Z)	**5.25 (0.006)**	0.99 (0.32)	1.41 (0.23)	0.96 (0.39)	0.26 (0.68)	**17.40 (0.001)**	0.18 (0.83)
Time to return	3.43 (0.04)	5.88 (0.02)	1.77 (0.18)	1.42 (0.25)	0.75 (0.47)	**14.6 (0.001)**	0.10 (0.91)

r is raw value, and Z is Z-score.

**Table 4:** Ordinal logistic regression parameters.

						Overall Model Test		
Models		Deviance	AIC	BIC	R^2^_McF_	χ^2^	df	*p*
1	ET	271.06	284.07	320.13	0.04	17.35	4	0.01
2	EDA	305.30	313.33	327.12	0.01	1.10	2	0.57
3	ECG	304.87	312.82	342.11	0.01	3.56	6	0.73
4	Br	385.11	412.53	434.28	0.03	12.69	5	0.02
**5**	**ET + Br**	**245.41**	**169.42**	**325.99**	**0.09**	**23.01**	**6**	**0.001**
6	ET + Br + ECG	244.41	162.83	341.21	0.11	31.55	12	0.001
7	ET + Br + EDA	281.52	301.52	333.99	0.08	23.86	8	0.002
8	ET + Br + ECG + SpO_2_	282.51	306.53	336.91	0.08	23.88	9	0.004
9	ET + Br + ECG + EDA	299.22	321.22	357.53	0.04	13.16	11	0.01
10	ET + Br + ECG + EDA + SpO_2_	277.81	303.83	356.86	0.04	32.58	15	0.005

ET is eye tracking parameters, EDA is electrodermal activity, Br is the breathing parameters, AIC is the Akaike information criteria, BIC is the Bayesian information criterion, R^2^_McF_ is McFadden’s R-squared and Df is degree of freedom.

**Table 5:** Ordinal logistic regression parameters (model 5).

						Confidence Interval	
Predictor	Estimate	SE	Z	*p*	Ratio	Lower	Upper
**Breathing rate**	**0.25**	**0.07**	**3.61**	**0.02**	**1.28**	**1.11**	**1.49**
Breathing variability (HFn)	−0.47	0.1	−4.80	0.05	0.93	0.89	1.00
Breathing variability (LFn)	0.37	0.16	1.83	0.07	1.60	0.96	2.60
Pupil size (Z-score)	0.47	0.63	1.81	0.07	1.60	0.98	2.63
**PDR Amplitude (Z-score)**	**−0.14**	**0.07**	**0.61**	**0.02**	**0.87**	**0.74**	**0.91**
PDR Latency (Z-score)	0.05	0.02	1.30	0.11	1.02	1.01	1.10
PDR Return	−0.04	0.12	-0.30	0.77	0.96	0.76	1.23

SE is the standard error.

**Table 6:** Experimental tasks configuration.

	Event Frequency (per min)			Details
	Low	Medium	High	
TRACK	Continue	Continue	Continue	Identical across all three levels
SYSMON	0.7	2.5	5.0	Only F1–F4 are used
RESMAN	0.7 failures	2.5 failures	5.0 failures	Target: Tanks A = 2500 units Tanks B = 1000 units
COMM	0.7	2.5	5.0	Target: 33% (low) or 25% (medium and high)
ODDBALL	12	12	12	20% target sound (identical across all 3 levels)
Task Overlap	No	No	Yes	Some stimuli can be presented at the same time

## Data Availability

Data Availability Statements are available after demand to the corresponding author.

## Supplementary Materials

The following supporting information can be downloaded at https://www.mdpi.com/article/10.3390/clockssleep6030024/s1, Figure S1: Correlations analysis between physiological parameters and NASA-TLX score during the four experimental conditions.

## Appendix A. Definitions and References for Physiological Parameters

**Table A1:** HRV parameters associated with mental workload.

Domain	Feature	Definition	References
**Time** **domain**	**RMSSD**	The square root of the mean of the squared successive differences between adjacent RR intervals	[73,74]
	**CVI**	Cardiac Vagal Index: index of cardiac parasympathetic function. Logarithm of the product of longitudinal (4*SD2) and transverse variability (4*SD1)	[75]
	**SDNN**	The standard deviation of the RR intervals	[6,73]
	**CVNN**	Coefficient of variation. The standard deviation of the RR intervals (SDNN) divided by the mean of the RR intervals (MeanNN)	[73]
	**pNN50**	The proportion of RR intervals greater than 50 ms	[73,74]
	**HTI**	Heart rate variability triangular index. Calculates the integral of the density of the R-R interval histogram divided by its height per 5 min	[74]
	**TINN**	Triangular interpolation. Baseline width of the RR intervals distribution obtained by triangular interpolation (approximation of the RR interval distribution)	[74]
**Frequency** **domain**	**HFn**	Normalized high frequency, obtained by dividing the high-frequency power (0.15 to 0.4 Hz) by the total powe.	[6,73]
	**LFn**	Normalized low frequency, obtained by dividing the low-frequency power (0.04 to 0.15 Hz) by the total power.	[6,73]
	**VLF**	Spectral power of very low frequencies (0.0033 to 0.04 Hz).	[6,73]
	**LF/HF**	Low-frequency power/high-frequency power.	[6,74]
**Entropy**	**ShanEN**	Basic measure of entropy (quantify the amount of information in a variable) $ShanEn=-\sum_{x\in X}p\left(x\right)\log_{2}p(x)$	[74,76]
	**ApEn**	Approximate entropy. Quantify the amount of regularity and the unpredictability of fluctuations over time-series data (complexity of physiological time series)	[74]
	**SampEn**	The conditional probability that two vectors that are close to each other form dimensions will remain close at the next m + 1 component.	[73,74]

**Table A2:** Respiratory parameters associated with mental workload.

Domain	Feature	Definition	References
**Time domain**	**Rate**	Mean respiratory rate	[6,9]
**Volume**	**Amplitude**	Mean respiratory amplitude.	[9]
**parameters**	**Inspiration**	Average inspiratory duration.	[9]
	**Expiration**	Average expiratory duration.	[9]
**Frequency domain**	**HFn**	Normalized high frequency, obtained by dividing the low-frequency power (0.004 to 0.15 Hz) by total power.	[57]
	**LFn**	Normalized low frequency, obtained by dividing the low-frequency power (0.15 to 0.4 Hz) by the total power	[57]

**Table A3:** Eye tracking parameters associated with mental workload.

Domain	Feature	Definition	References
**Pupil size**	**Raw value**	Mean pupil size	[15,54,77]
	**Z-score**	(raw value—mean baseline value)/baseline standard deviation	
**Pupil dilation**	**Amplitude**	The amplitude of phasic dilation of the pupil peaking 1–1.6 s after the auditory stimulus	[78,79]
**response (PDR)**	**Latency**	Latency of phasic dilation of the pupil peaking 1–1.6 s after the auditory stimulus	
	**Time Return**	Average value phasic dilation of the pupil peaking 1.6–2 s after the auditory stimulus	

**Table A4:** EDA parameters associated with mental workload.

Domain	Feature	Definition	References
**Temporal**	**Tonic activity**	Also known as SCL, refers to the smooth and gradual changes in the EDA response signal. Express in amplitude, μS	[71,79]
	**Phasic activity**	Also known as SCR, refers to the rapid/sudden changes in the EDA response. Express in amplitude, μS	[71]
