# Supplementary material for: Combined Effects of Moderate Hypoxia and Sleep Restriction on Mental Workload
Source: Clocks Sleep. 2024 Jul 23;6(3):338–58. doi: 10.3390/clockssleep6030024 (PMC11348049; doi:10.3390/clockssleep6030024)
Supplement: Supplementary file 1 [file clockssleep-06-00024-s001.zip › clockssleep-3043906-supplementary.pdf]

	Habitual Sleep Normoxia	Habitual Sleep Hypoxia	Sleep restriction Normoxia	Sleep restriction Hypoxia
	NASA TLX	NASA TLX	NASA TLX	NASA TLX
Heart rate	-0.10	-0.13	-0.10	-0.05
HRV_SDNN	0.31	0.18	-0.29	0.05
HRV_RMSSD	0.38	0.22	-0.23	-0.07
HRV_PNN50	0.23	0.22	0.26	0.07
HRV_CVI	0.28	0.21	0.33	0.21
HRV_HFn	0.09	0.17	-0.08	-0.13
HRV_VLF	0.21	0.16	0.01	0.16
HRV_ShanEn	-0.07	-0.01	0.08	-0.21
HRV_SampEn	-0.01	0.07	0.01	-0.09
EDA_Tonic	-0.31	-0.36	-0.57	-0.48
**Breathing rate**	0.26	0.27	0.27	-0.22
Breathing variability (LFn)	0.09	0.09	0.26	-0.16
Breathing variability (HFn)	0.13	-0.18	-0.27	0.11
Mean pupilar size (r)	0.36	0.07	0.13	-0.24
**Blinks number**	0.41	0.39	0.29	0.21
**Blinks duration**	0.31	0.49	0.21	0.31
Pupil Phasic size (r)	-0.07	-0.14	0.12	-0.06
**PDR Amplitude (Z)**	-0.32	-0.39	-0.21	-0.11
PDR Latency	0.62	0.26	0.18	0.19
**PDR Time return**	0.11	-0.19	-0.11	-0.15

Pearson coefficient R	Corrected p
> |0.60|	< 0.0001
> |0.50|	< 0.001
> |0.40|	< 0.01
> |0.30|	< 0.02
> |0.20|	< 0.05

Figure S1. Correlations analysis between physiological parameters and NASA TLX score during the 4 experimental conditions. Only parameters significantly correlated ($r > |0.20|$, $p < 0.05$) with MATB-II tracking performance (the RMSD value) in habitual sleep and normoxia are presented.
